# Independent EEG Sources Are Dipolar

**DOI:** 10.1371/journal.pone.0030135

**Published:** 2012-02-15

**Authors:** Arnaud Delorme, Jason Palmer, Julie Onton, Robert Oostenveld, Scott Makeig

**Affiliations:** 1 Swartz Center for Computational Neuroscience, University of California San Diego, La Jolla, California, United States of America; 2 Centre de recherche Cerveau et Cognition, Paul Sabatier University, Toulouse, France; 3 Donders Institute for Brain, Cognition and Behaviour, Radboud University, Nijmegen, The Netherlands; 4 CERCO, CNRS, Toulouse, France; 5 Naval Health Research Center, San Diego, California, United States of America; University of British Columbia, Canada

## Abstract

Independent component analysis (ICA) and blind source separation (BSS) methods are increasingly used to separate individual brain and non-brain source signals mixed by volume conduction in electroencephalographic (EEG) and other electrophysiological recordings. We compared results of decomposing thirteen 71-channel human scalp EEG datasets by 22 ICA and BSS algorithms, assessing the pairwise mutual information (PMI) in scalp channel pairs, the remaining PMI in component pairs, the overall mutual information reduction (MIR) effected by each decomposition, and decomposition ‘dipolarity’ defined as the number of component scalp maps matching the projection of a single equivalent dipole with less than a given residual variance. The least well-performing algorithm was principal component analysis (PCA); best performing were AMICA and other likelihood/mutual information based ICA methods. Though these and other commonly-used decomposition methods returned many similar components, across 18 ICA/BSS algorithms mean dipolarity varied linearly with both MIR and with PMI remaining between the resulting component time courses, a result compatible with an interpretation of many maximally independent EEG components as being volume-conducted projections of partially-synchronous local cortical field activity within single compact cortical domains. To encourage further method comparisons, the data and software used to prepare the results have been made available (http://sccn.ucsd.edu/wiki/BSSComparison).

## Introduction

Brain-generated EEG data are generally considered to index synchronous aspects of local field potentials surrounding radially-arrayed cortical pyramidal cells [1], [2]. There are strong biological reasons to believe that under favorable circumstances ICA should separate signals arising from local field activities in physically distinct, compact cortical source areas: First, short-range (<100 µm) lateral connections between cortical neurons are vastly more dense than longer-range connections [3], [4], while inhibitory and glial cell networks have no long-range processes [3]. Also, thalamocortical connections that also play a strong role in cortical field dynamics [5], [6] are predominantly radial. For these reasons, synchronization of cortical field activities within sparsely connected distributed domains should be much weaker than whole or partial synchronization of field activity within compact domains supported by short-range anatomic connections. Thus, cortical field potentials contributing to scalp EEG should arise largely from near-synchronous field activities within cortical ‘patches’ whose net far-field signals are near-instantaneously volume conducted to and linearly summed at EEG scalp electrodes.

Emergence of near-synchronous field activity within small cortical domains has been observed and modeled *in vivo* (‘phase cones’) [7], *in vitro* (‘neuronal avalanches’) [8], [9] and *in silico* [10]. The net far-field projection of such a cortical patch will nearly equal that of a single ‘equivalent’ current dipole located near and typically beneath the center of the generating patch [11], [12]. In practice, we have observed that linear decomposition of high-density scalp EEG data by Independent Component Analysis (ICA) may return up to dozens of maximally independent component processes whose scalp maps are generally compatible with their generation in such a cortical patch [13], [14], [15], [16], [17]. This suggests an approach to comparing the relative biological plausibility and utility for EEG source separation of the many ICA and other blind source separation (BSS) algorithms that have been introduced in the last two decades.

### BSS/ICA sources

BSS and, in particular, ICA methods are now widely used for separating artifacts from scalp-recorded electroencephalographic (EEG) and related data [18], [19], [20], [21], [22] and, increasingly, to separate and study brain source activities [13], [14], [23], [24], [25], [26]. ICA identifies signals in recorded multi-channel data mixtures whose time courses are maximally independent of one other and in this sense contribute maximally distinct information to the recorded data. Instead of directly addressing the general EEG inverse problem of determining the time courses *and* spatial distributions of cortical (and other) source areas of recorded scalp signals using an electrical forward head model (estimating the projection weights of all possible sources to the scalp sensors), ICA directly models *What* distinct signals are contained in the volume-conducted scalp data, and returns the relative projection strength of each maximally independent source to the scalp sensors, thereby also greatly simplifying the problem of determining *Where* in the brain each EEG source signal is generated [12], [24].

### Non-brain sources

Scalp-recorded EEG data also include non-brain or ‘artifact’ signals that are linearly mixed with brain EEG source activities at the scalp electrodes. ICA has been found to efficiently separate out several classes of spatially stereotyped non-brain signals: scalp and neck muscle electromyographic (EMG) activities, electro-oculographic (EOG) activities associated with eye blinks [20], saccades, and ocular motor tremor [15] as well as electrocardiographic (ECG) signal and single-channel noise produced by occasional loose connections between electrodes and scalp. Spatially non-stereotyped artifacts associated with irregular scalp maps (for example, artifacts produced by extreme participant movements) cannot be parsed by ICA into one (or a few) component(s), so these are best removed from the data before decomposition.

### Decomposition differences

Though ICA algorithms all have the same root goal [27] and generally produce similar results when used to unmix idealized source mixtures, since EEG brain and non-brain source signals are likely not perfectly independent and different algorithmic approaches to maximizing independence differ, different ICA algorithms may return somewhat different results when applied to the same EEG data. Unlike most ICA algorithms that attempt to minimize instantaneous dependence, some BSS algorithms attempt to reduce redundancy between lagged versions of the data. To date, the three ICA/BSS algorithms applied most often to EEG data are likely extended Infomax ICA [27], [28], so-called FastICA [29], and Second-Order Blind Identification (SOBI) [30]. Computer code for these and a variety of other proposed ICA and BSS algorithms are readily available, making of interest a comparison of their effectiveness for EEG data decomposition.

### Comparing decompositions

To date, however, suitable measures have not been demonstrated for comparing the components returned by different ICA/BSS algorithms applied to actual (as opposed to simulated) EEG data for which ‘ground truth’ source signals and scalp projections are not available. In particular, components produced by ICA decompositions that minimize mutual information between simultaneously recorded signal values have not been much compared to components produced by BSS algorithms that simultaneously minimize component signal redundancy at multiple time delays [16], [21].

Here, we use three measures – the amount of mutual information reduction (MIR) between the recovered component time courses relative to the recorded data channels (in kbits/sec), the mean remaining pairwise mutual information (PMI) between pairs of component time courses (in kbits/sec) [31], and the ‘dipolarity’ of the decomposition defined as the number of returned components whose scalp maps can be fit to the scalp projection of a single equivalent dipole with less than a specified error threshold (specified as percent residual variance). We applied these measures to 71-channel EEG data we had collected from 14 subjects (roughly 300,000 time points for each) as they performed a modified Sternberg visual working memory task [25] (see Methods).

The motivation for the first two measures (MIR and PMI) is clear; they test how well the results of the decomposition approach the instantaneous independence objective. MIR, introduced here, is a direct (and as we show, easily computed) measure of the statistical distinctness of the activities of the resulting components, the absence of dependency entailing, in particular, suppressing the strong linear mixing of EEG signal source signals by common volume conduction to the electrodes. PMI is a partial measure of MIR that can also be directly (though less efficiently) computed from the data. MIR takes into account multi-component dependencies whereas PMI only considers pairwise dependencies. Both can be said to index the relative success of ICA/BSS in finding component processes with fixed scalp projection patterns and near-independent time courses whose projections sum to the data.

The motivation for the third measure (dipolarity) is the assumption that brain and non-brain EEG sources have spatially fixed source locations and orientations, as well as temporally distinct, independently varying time courses. This assumption is reasonable at least for scalp muscle, ocular, and electrode artifact signals and, as described above, for many cortical source processes as well, whose volume-conducted potentials recorded at the scalp represent far-field projections of signals each generated within a compact patch of coherent cortical field activity (e.g., within a cortical ‘phase cone’ [32] or ‘neuronal avalanche’ sequence [8], [9]). While this may not perfectly describe all brain EEG sources, the total numbers of such sources separated by these decompositions is of interest since they allow interpretation as locally synchronous field signals from distinct (and more simply localizable) cortical areas.

Here, we show that these three rather different measures, the first two considering only the component time courses and the third only the component scalp maps, are redundant, as they similarly rank-order decomposition differences for a large subset of available ICA/BSS methods applied to actual high-density EEG data. This result is compatible with a model of many EEG signal sources as originating in partially synchronous local field activity across a cortical patch or spatially fixed non-brain artifact generator and, we believe, further supports the utility of ICA decomposition for identifying physiologically and functionally distinct sources of high-density EEG data.

## Results

### Mutual information reduction (MIR) and dipolarity

Table 1 shows results for all 22 algorithms tested, sorted by their efficiency in temporal information separation, as measured by total mutual information reduction (MIR) in kbits/sec, plus a summary measure of biological plausibility, the percentage of (near-dipolar) component equivalent dipoles whose scalp maps differed less that 10% by variance from the best-fitting equivalent dipole model (ND10%). Components identified by the AMICA decomposition [33] produced the largest mutual information reduction – about 43.1 kbits/sec (180 bits per frame or time point) – and the most near-dipolar components (on average 34/71 or 48%) with less than 10% residual scalp map variance from the projection of the best-fitting equivalent dipole. Results for Extended Infomax, Pearson, and (super-Gaussian) Infomax approached those of AMICA, with nearly the same mutual information reduction (within half a bit per frame or time point) and about 40% of their component maps near-dipolar.

**Table 1:** Mean mutual information reduction and ‘dipolarity’ measures for each algorithm across 13 data sets.

Algorithm (MATLAB function)	MIR (kbits/s)	ND 10%	Origin^1^
AMICA	43.12	48.6	EEGLAB 6.1
Infomax (runica)	43.07	41.6	EEGLAB 4.515
Extended Infomax (runica)	43.02	43.8	EEGLAB 4.515
Pearson	43.01	42.6	ICAcentral (6)
SHIBBS	42.74	34.1	ICAcentral (5)
JADE	42.74	33.9	EEGLAB 4.515
FastICA^2^	42.71	35.5	ICAcentral (2)
TICA	42.68	34.1	ICALAB 1.5.2
JADE_OPT. (jade_op)	42.64	31.0	ICALAB 1.5.2
SOBI^4^	42.51	24.5	EEGLAB 4.515
JADE_TD (jade_td)	42.47	28.9	ICALAB 1.5.2
SOBIRO^4^ (acsobiro)	42.44	26.4	EEGLAB 4.515
Sphering	42.34	87.0	EEGLAB 4.515
FOBI	42.31	26.5	ICALAB 1.5.2
EVD24	42.30	25.4	ICALAB 1.5.2
EVD	42.19	23.7	ICALAB 1.5.2
icaMS^3^	42.18	14.0	ICA DTU Tbox
AMUSE	42.14	11.9	ICALAB 1.5.2
PCA	41.86	4.4	EEGLAB 4.515
SONS	41.76	37.5	ICALAB 1.5.2
eeA	39.98	27.4	ICAcentral (8)
ERICA	38.78	39.7	ICALAB 1.5.2

1 EEGLAB (sccn.ucsd.edu/eeglab); ICAcentral (tsi.enst.fr/icacentral); ICALAB (bsp.brain.riken.go.jp/ICALAB); ICA DTU Toolbox (mole.imm.dtu.dk.toolbox/ica). Numbers in parentheses to the right of the ICAcentral source label indicate the entry in the ICACentral.org database; other numbers in this position give the toolbox version used.

2 A symmetric approach to optimizing the FastICA weights returned similar results.

3 By default not using pre-whitening.

4 The time lag used was 100 samples, which is supposed to be optimal for EEG data.

The leftmost column gives the algorithm used (and when ambiguous, the MATLAB function in parentheses). The second column (Mutual Information Reduction, MIR, in kilobits per second) indicates the excess mutual information remaining among the component time courses, compared to the component time courses of the most efficient algorithm tested (AMICA). The third column (near-dipolar percentage, ND10%) indicates the percentage of returned components whose scalp maps had less than 10% residual variance from the scalp projection of the best-fitting single equivalent dipole model. The fourth column (Origin) indicates the online source repository from which the MATLAB source code was obtained (see footnotes).

Component time courses returned by the BSS algorithm SOBI produced less MIR (610 bits per second less MIR than AMICA), ranking its output midway between the most efficient ICA method (AMICA) and the least efficient (PCA, producing about 1260 bits per second less MIR than AMICA). The mean percentage of near-dipolar (ND) components for SOBI (24%) was also midway between the highest (AMICA) and lowest (PCA) results. While completely uncorrelated by definition, the sets of PCA components were not as highly independent, retaining higher-order dependencies.

**Figure 1:**
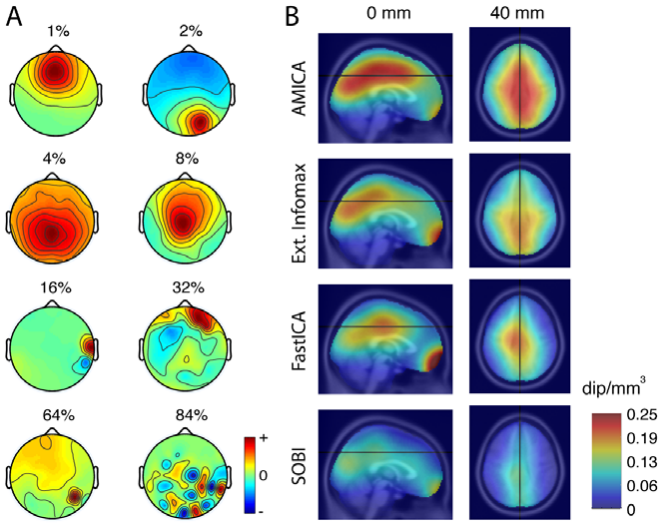
Component dipolarity and equivalent dipole density. **A**. Example component scalp maps more (top) to less (bottom) resembling the projection of a single equivalent dipole. Eight interpolated independent component (IC) scalp maps with progressively more difference from the projection of the best-fitting equivalent dipole (ED) (percent of residual variance (r.v.) indicated for each map). All but the bottom-right component scalp map are from AMICA decompositions. 1^st^ row: (left) frontal midline IC with prominent theta band activity; (right) right parietal IC with a more tangentially oriented ED model and prominent alpha band peak. 2^nd^ row: (left) central parietal IC likely reflecting coupled field activity in adjacent left and right medial cortex; (right) more anterior midline IC. 3^rd^ row: (left) IC accounting for EMG activity of a right post-auricular muscle; (right) IC of uncertain origin. 4^th^ row: (left) IC accounting for electrode noise at the most affected (red) scalp electrode; (right) high-order PCA component with a characteristic ‘checkerboard’ scalp map unlike the projection of any single ED. **B**. Mean (left) medial sagittal and (right) axial (z = 40 mm) densities of near-dipolar (r.v.< = 5%) component equivalent dipoles across all data sets, from four (indicated) decomposition methods.

**Figure 1A** shows exemplar scalp maps for seven (AMICA) components with successively higher amounts of equivalent dipole residual variance (from 1% to 64%), plus one component (lower right, a typical PCA high-order ‘checkerboard’-like map with still higher r.v. = 84%) clearly incompatible with the projection of single brain equivalent dipole. **Figure 1B** shows, for four decomposition algorithms, the mean density (in dipoles per mm^3^) of equivalent dipoles for components with near-dipolar (r.v.<5%) scalp maps in two brain slices. The figure reflects the fact that applied to these data sets AMICA returned a larger number of near-dipolar components than extended infomax ICA, FastICA, or SOBI. For this reason, we used AMICA as a standard for exploration of the similarities and differences between components returned by the other decompositions. Source densities for PCA and sphering decompositions are not shown: Very few of the PCA component scalp maps were near-dipolar – for example on average only 3 of 71 at the 10% residual variance (r.v.) cutoff, and those mainly dominated by large eye artifacts. A much larger number of ‘sphering’ components (87%) had near-dipolar scalp maps, as is guaranteed by the nature of the sphering decomposition (see Methods). However, as the best-fitting single-dipole models for these sphering component maps are near-radial to the scalp surface and located below each of the scalp channels, respectively, rather than indicating the location and orientation of an actual EEG source.

### General component similarity

Next, we made a preliminary assessment of whether the best-known ICA and BSS algorithms generally returned similar components. To do this, we first selected from the AMICA decomposition of one participant's data, by visual inspection of scalp maps, time courses, and power spectra, representative components of seven types – central occipital alpha band (near 10-Hz) activity, frontal midline theta band (near 5-Hz) activity, eye blink artifacts, left and right mu rhythm activities (near 10 Hz, with prominent harmonics), lateral saccade artifacts, and electromyographic (EMG) activity from a right forehead muscle. We then found the components from four other decompositions of the same data set (Infomax ICA, FastICA, SOBI, and sphering) whose scalp maps were most correlated to the seven representative AMICA component maps.

**Figure 2:**
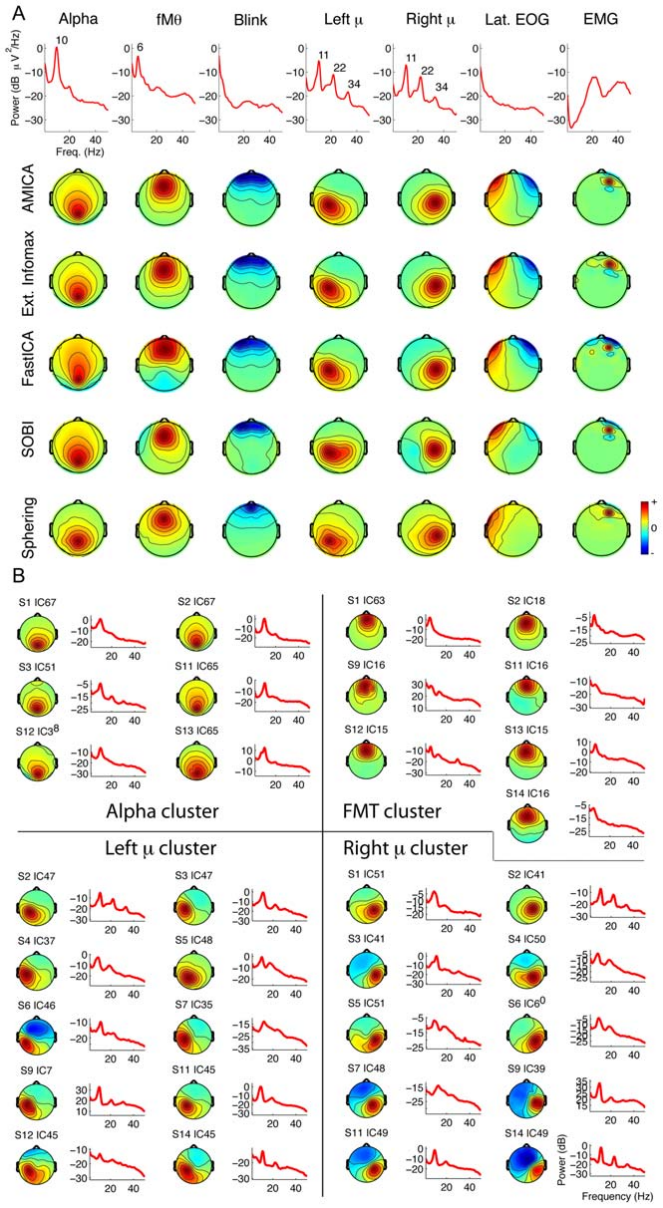
ICA algorithms return similar dipolar components. **A**. The top rows show activity spectra and scalp maps for seven AMICA components from one subject accounting respectively for eye blinks, lateral eye movements (EOG), and right frontal scalp muscle activity as well as posterior alpha band, central mu rhythm, and frontal midline theta activity. Lower four rows show scalp maps of best-matching components by Extended Infomax ICA, FastICA, SOBI, and sphering decompositions of the same subject data, e.g., those with highest absolute scalp map correlations to the respective AMICA components. Note the resemblance of the scalp maps in each case for AMICA and Extended Infomax, and the differences between the AMICA and sphering component maps. **B**. Scalp maps and mean activity spectra of four clusters of similar AMICA components from 6–10 different data sets from different subjects, isolated by visual inspection of component scalp maps and mean activity spectra and accounting respectively for central occipital alpha, frontal midline theta, and left and right mu rhythm activities.

Results in **Figure 2A** show the scalp maps of these ‘best-matching components’ strongly resembled those returned by AMICA. Note, however, the relative inability of sphering (bottom row, two rightmost columns) to find component maps with tangentially-oriented equivalent dipole models (lateral eye movement artifacts and right frontal scalp muscle activity). Also, as expected the eye blink artifacts (third column) clearly separated by all but the sphering decomposition (bottom) clearly could be better fit with dual-symmetric equivalent dipole models (one dipole for each eye), though here for simplicity we chose to ignore the presence of such components in defining decomposition ‘dipolarity.’ These representative components are present in most datasets.

**Figure 3:**
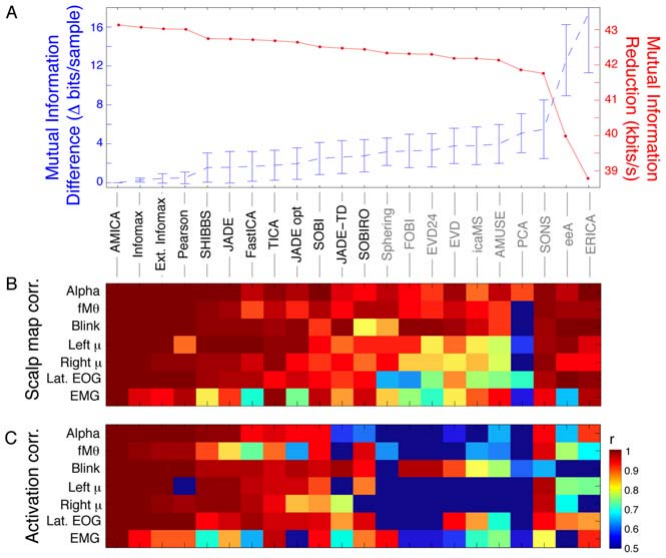
Correlations of component scalp maps and time courses across algorithms for the seven identified component types. **A**. (Blue trace) Mean (±std. deviation across all data sets) additional mutual information remaining in the time courses of components returned by each of the 22 decomposition algorithms relative to AMICA decompositions of the same data, sorted left to right by overall degree of mutual information reduction (MIR, red trace). Names of the 12 algorithms returning components with more mutual information reduction (MIR) than simple sphering (bottom left) are lettered in black. **B**. Mean correlations between seven representative AMICA component scalp maps from one participant (same as top row in **Figure 2**) and the seven components with best absolute scalp map correlations to these returned by the other decompositions of this subject's data. **C**. Mean correlations between the independent component activation time courses for the same component pairs as in B. Although the time course correlations are generally lower than scalp map correlations, the two patterns of results are similar, with (leftmost) decompositions effecting the most mutual information reduction returning components whose scalp maps and activities generally strongly resemble the selected AMICA components.

Next, we found clusters of AMICA components from the other data sets most similar in location and dynamics to each of the original seven components; the four component clusters accounting for non-artifact brain sources are shown in **Figure 2B**. To further quantify relationships between components returned by the different decompositions, we computed the mean absolute correlations between scalp maps and activity time courses of components from all the algorithms that best matched the seven selected AMICA components shown in **Figure 2A**. Results are shown in **Figure 3**.

For all of the twelve algorithms that reduced average pairwise mutual information more than sphering (the highlighted algorithm labels in **Figure 3A**), absolute correlations between the AMICA component scalp maps and the best-matching component scalp maps were mostly above 0.9 (**Figure 3B**), as were most of the time course correlations (**Figure 3C**). For comparison, **Figure 3A** shows the MIR (red) and difference in MIR from AMICA (blue) for each algorithm. For unknown reasons, on this particular type of high dimensional EEG data, three ICA algorithms (SONS, eeA, and ERICA, rightmost columns) were markedly less efficient in reducing mutual information overall, although the scalp maps of their best-matching components also generally resembled (r>0.9) those of the seven selected AMICA components (see Discussion). As the time courses of the best map-correlated sphering components (**Figure 3**, middle column) were not well correlated with those returned by the ICA algorithms, we omitted sphering from further method comparisons.

### Decomposition dipolarity

**Figure 4:**
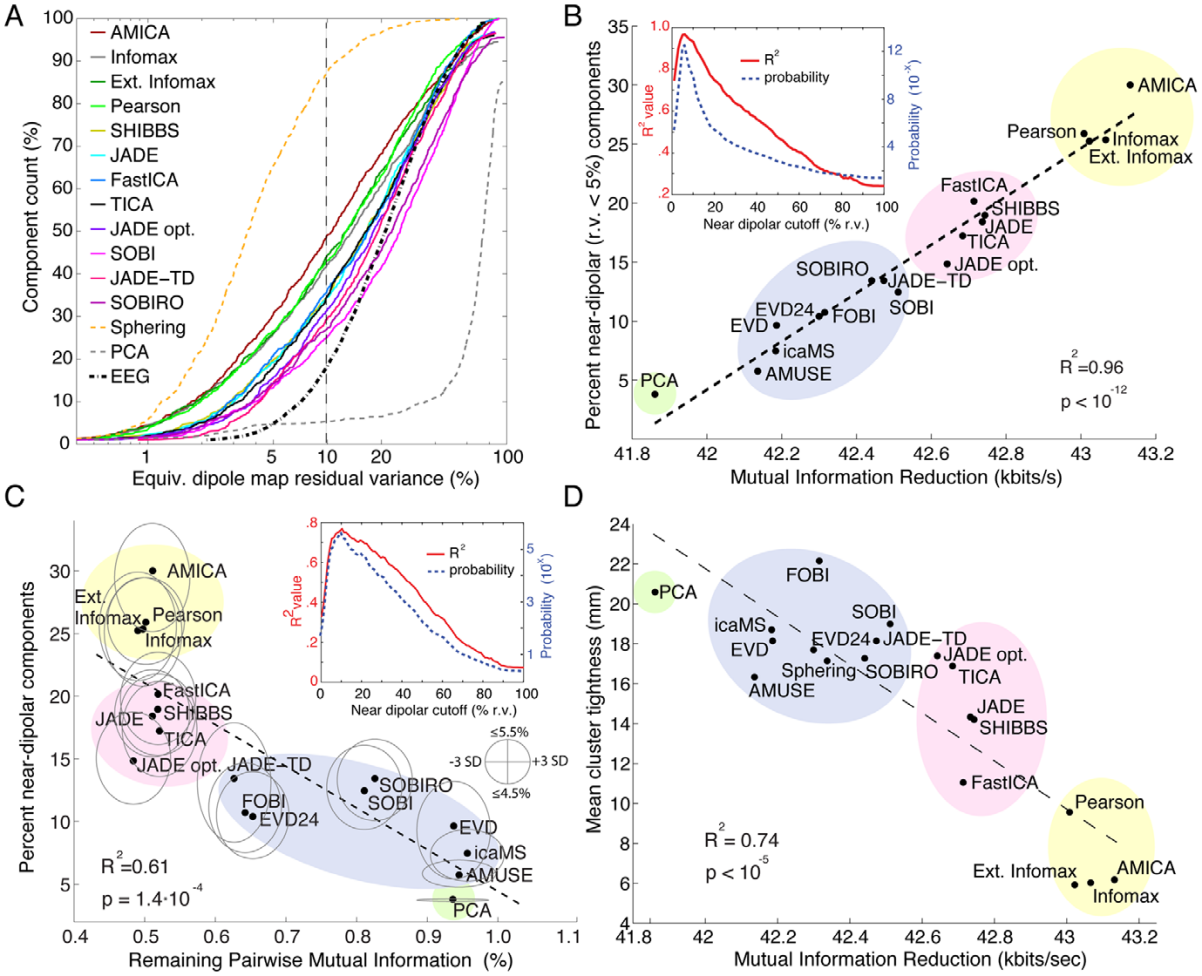
Decompositions that reduce total mutual information in the component time courses also return more components with near-dipolar scalp maps. **A**. Cumulative mean percentage of components returned by each blind source separation algorithm sorted by percent scalp map residual variance (r.v.) remaining after subtracting the best-fitting single equivalent dipole model. The key lists the decomposition methods in order of their mean number of near-dipolar components (e.g, having scalp map r.v.< = 10%). Note the topmost yellow dashed trace (sphering), the bottommost trace (PCA, principal component analysis), and the black dashed trace (mean cumulative dipolarity of 71 sample EEG scalp maps randomly selected from each dataset). **B**. (Ordinate) percentage of components with strongly dipolar scalp maps (r.v.< = 5%), plotted against (abscissa) mean mutual information reduction (MIR) for 18 of the algorithms. The dashed line shows the linear regression (R^2^ = 0.96, p<10^−12^). Figure inset: (red trace) Probability that MIR varies linearly with the proportion of near-dipolar components, and (blue) proportion of variance accounted for by the linear fit, as functions of ‘near-dipolar’ r.v. threshold. Both variables peak at a ‘dipolar’ residual variance cutoff of 6%. The 18 decomposition methods form four groups (colored oval highlights added manually to group algorithms by type; see Methods). The computed standard deviations of these MI values are too small to be represented. **C**. Percentage near-dipolar components (with scalp map r.v.< = 5%) as a function of mean percentage of channel pairwise mutual information (PMI) remaining between component time courses. Ellipses around each data point indicate, on the horizontal axis, 3-std. dev. confidence bounds for each component PMI calculation. Note: the PMI standard error of the mean (SEM) confidence region is ∼180 times more narrow. The heights of the ovals show the range of decomposition ‘dipolarity’ values for neighborhood r.v. cutoff values between 4.5% and 5.5% ; other details as in B. **D**. For the seven identified component clusters for each decomposition method (as in **Figure 2**), component cluster tightness (CCT) was defined as mean distance from each component equivalent dipole to the method cluster dipole centroid. As expected, mean CCT was smallest for AMICA. Across all decomposition methods, the relationship between cluster tightness and MIR again had a near linear trend (r^2^ = 0.74). Thus, in general decompositions producing more MIR also returned components of seven identified types with more consistent equivalent dipole locations across subjects.

Next, we computed the resemblance of the scalp maps of each component returned by the remaining decomposition methods to the scalp projection of a best-fitting single equivalent dipole. **Figure 4A** shows the cumulative distributions of percent residual scalp map variance left unaccounted for by the equivalent dipole model for all components returned by the remaining 18 ICA/BSS algorithms. The cumulative percentage of near-dipolar components can be read from the ordinate for each residual variance cutoff on the abscissa. The three black/grey dashed traces allow comparison with quite different results of sphering (top), of PCA whitening (bottom), and of attempting to fit equal numbers of randomly selected raw, single time-point EEG data scalp maps with a single equivalent dipole model (center). Note the largely increased number of ‘near-dipolar’ ICA component maps compared to the raw channel EEG data. SOBI and the other time-dependent algorithms did not return as many near-dipolar components as the natural gradient-based ICA algorithms, which were led by AMICA, which returned on average 34 (of 71) such components at an (arbitrary) 10% residual variance dipolarity threshold.

### Mutual information reduction (MIR) versus dipolarity

Next, we asked whether the algorithms that better reduced the mutual information present in the raw scalp channel time courses also returned more components with near-dipolar scalp maps. **Figure 4B** plots, for the remaining 18 decompositions, the relationship between mean algorithm MIR and algorithm dipolarity, here defined as number of components with single equivalent dipole model residual variance below a stricter threshold (r.v.≤5%). The figure reveals a surprisingly strong and highly significant linear relationship (r^2^ = 0.96) between the mean increase in the independence of the component time courses (in MIR kbits/second) and the number of returned components with near-dipolar scalp maps.

The inset to **Figure 4B** shows the r^2^ and probability (by t-test) of the regression line for residual variance dipolarity thresholds between 2% to 99%. The inset shows that the positive slope of the regression line in the main figure is significant by t-test (at p<10^−4^) at residual variance (r.v.) thresholds from 2% to over 20%, with the linear fit accounting for more than 96% of algorithm variance at its peak (r.v.< = 6%). The 6% r.v. threshold might not be far above the minimum r.v. error compatible with our use, here, of a simple best-fitting spherical head model and standardized electrode locations to compute the scalp map projections of the equivalent dipoles; using a best-fitting spherical head model instead of an individual head model built from a subject MR head image adds on average a 3% r.v. error (Z. Akalin Acar, personal communication). Components with scalp map r.v.< = 6% may thus be quite consistent with the projection of a single equivalent dipole (though this fact alone does not necessarily rule out source geometries other than a single compact cortical patch domain). In brief, **Figure 4B** indicates that ***more independent linear EEG decompositions include more near-dipolar components***, and that ***this monotonic relationship is on average nearly linear across a large number of ICA and BSS algorithms from PCA to Infomax ICA and AMICA***.

### Pairwise mutual information (PMI) and dipolarity

Plotting algorithm dipolarity, defined as the mean number of returned components with near-dipolar (here, r.v.<5%) scalp maps, against the mean percentage of mutual information remaining between *pairs* of component time courses, compared to pairwise mutual information between EEG scalp channels (**Figure 4C**), revealed a similar linear trend. The more completely the decomposition eliminates pairwise mutual information, the more near-dipolar components are separated by the decomposition. However, the converse is not true; pairwise mutual information measures only account for a portion of total mutual information, which may also obtain exclusively within subspaces of *more than two* signals. Though linear trend (r^2^ = 0.71) for PMI is not as strong as for total MIR, the residual variance cutoff at which this linear trend is maximum is near equivalent (cf. **Figure 4B**).

### Mutual information and equivalent dipole location

For the four brain component types shown in **Figure 2B**, we found the component returned by each of the 18 algorithms for each of the 13 datasets whose scalp map most closely resembled that of the exemplar AMICA component (**Figure 2A**, top row). We defined the mean cluster ‘tightness’ of the cluster of these 13 components as the mean distance from each cluster component equivalent dipole model to the location of the dipole cluster centroid. The resulting mean cluster tightness for each decomposition algorithm is shown in **Figure 4D** plotted against its mean MIR. As expected, cluster tightness was smallest for AMICA since the clustered components from the other decompositions were those with best (but imperfect) scalp map correlations to the exemplar AMICA components. However, the relationship between component cluster tightness (in root mean square mm) and the mutual information reduction (MIR) achieved by the other algorithms again had a strong linear trend (r^2^ = 0.74). Thus, on average algorithms that returned more near-dipolar components (**Figures 3** **and** **4**) also returned components (of the four selected types) whose equivalent dipole locations, across participants, were more consistent.

**Figure 5:**
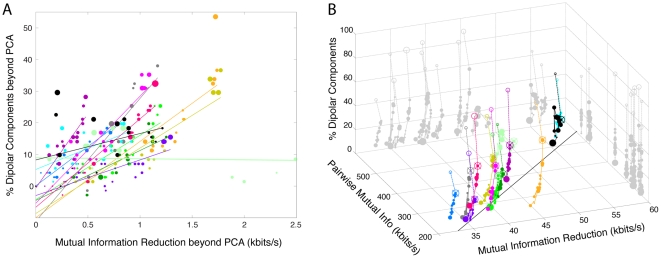
Channel pair-wise mutual information, mutual information reduction, and dipolarity for each dataset and algorithm. **A**. For each data set and decomposition, mutual information reduction versus difference in the percentage of near-dipolar components (less than 5% residual variance) and the (nearly always lower) percentage produced by PCA. The size of the disk markers is proportional to the number of components whose scalp maps are near the 5% r.v. threshold (in the range 4.5% to 5.5%). Colors group results for each dataset. For each dataset, a linear fit based on all 18 decompositions (excluding sphering) is shown by a straight line. Best-fitting lines for all datasets but one (light green) have a positive slope as in **Figure 4B** (p<10^−5^ by two-tailed parametric unpaired t-test; df = 12). **B**. The percentage of dipolar components (vertical axis) in each decomposition, versus (left-going axis) the initial mutual information in the channel data for each data set as estimated by total channel pairwise mutual information (PMI), and (right-going axis) the overall reduction in mutual information (MIR) effected by the decomposition. The linear trend across datasets (indicated by the best-fitting line drawn on the ‘floor’ of the plotting box) shows that as might be expected, the more (pairwise) mutual information in the channel data, the more mutual information was removed by all decompositions. Results for the 18 algorithms in **Figure 4**, plus sphering, are connected in order of mean mutual information reduction (MIR) in **Figure 4A**. Results of AMICA decomposition (crossed circles) are connected by a dashed line to those for sphering (empty circles). Sphering produced more near-dipolar components that the other ICA/BSS algorithms, but produced less mutual information reduction than ICA algorithms, while as expected returning components having scalp maps centered on each electrode location (compare Fig. 2A).

To confirm the consistency of these findings, **Figure 5A** plots, for each data set and decomposition algorithm, the number of near-dipolar components (r.v.< = 5%) returned versus mutual information reduction (MIR) produced by the decomposition. Here colors group results for each dataset. Dipolarity is positively related to MIR for 12 of the 13 data sets. The mean r^2^ value of the linear fit, across all datasets, is r^2^ = 0.64±0.29 (p<0.00001 by two-tailed unpaired t-test, df = 12).

**Figure 5B** plots decomposition dipolarity for each data set versus MIR and total scalp-channel PMI before decomposition. Results for the 18 algorithms (plus sphering) are connected by line segments in the same order as in the mean results shown in **Figure 4A** (i.e., from PCA to AMICA), with dashed lines connecting the AMICA results (crossed circles) and ‘sphering’ (open circle) results. **Figure 5B** again shows that one of the 13 data sets (colored light green) is an outlier for which pre-decomposition PMI is relatively high, and that MIR has no strong relation to the ‘dipolarity’ of the decompositions. The (vertical) orders of the decomposition results for the other 12 data sets consistently resemble the mean results shown in **Figure 4B**. The panel also shows that, as might be expected, the amount of mutual information reduction for each data set is roughly proportional to the original amount of channel-pair PMI in the data (as indicated by the linear trend plotted on the ‘floor’ of the plotting box). Note that the total mutual information in the channel data, though itself infeasible to compute and not shown here, can be expected to be smaller than the total PMI (here about 300 kbits/s) since much of the mutual information between different channel pairs may actually reflect common higher-order dependencies that are counted multiple times in calculating total PMI. In turn, total mutual information in the channel data must be larger than the MIR (here near 40 kbits/s).

## Discussion

Use of ICA to remove distinct sources of artifact data from EEG and other neuroimaging data is increasingly widely accepted [19], [20], [21], [22] and its use to isolate and characterize cortical sources increasing [13], [14], [16], [17], [24], [34]. Although many researchers may have been curious about the relative advantages and underlying validity of applying one or other ICA or BSS decomposition to their EEG data, to date there has been little head-to-head comparison of the results of these algorithms, principally because ‘ground truth’ knowledge of the sources of EEG data is not yet available (e.g., from high-definition recordings), and their statistics are thus also difficult to simulate accurately.

Physiologically, EEG signals originating within the brain are assumed mainly to be associated with near-synchronous field activities within a connected patch (or two densely connected patches) of cortical pyramidal cells sharing a common alignment near-perpendicular to the cortical surface [1]. In the absence of local coherence in cortical neural field activity, potentials from the countless cortical field microdomains must tend to cancel each other at the scalp – in this case *no* far-field cortical signals can be recorded at the scalp. Local cortical field activity that is partly or wholly coherent across a compact cortical patch, on the other hand, is volume-conducted to the scalp electrodes as far-field potential.

While spatiotemporal dynamics of local cortical field synchronies have not yet been fully observed, investigations using small electrode grids have modeled such field patterns as ‘phase cones’ [32] or as ‘neuronal avalanche’ events [8], [9] and mathematical cortical models have pointed to the possible importance of stochastic network resonance in producing measurable local field potentials [35], [36]. Each scalp EEG channel represents the time course of potential difference between an electrode pair (often transformed *post hoc* into the ‘average reference’ potential difference between each scalp electrode signal and the average potential across all the electrodes). Scalp EEG channels record differently weighted sums of the far-field signals that reach them from all brain and non-brain sources, plus any near-field electrode noise generated at the electrode/skin interface.

The high anatomic bias in cortical connectivity toward local (<100 µm) connections, as well as the primarily radial connectivity between cortex and thalamus, support the concept that the far-field signal emerging from one domain (island, patch) of local spatiotemporal field synchrony should be typically *predominantly* or *nearly* independent from any other such signal arising elsewhere in cortex (with exceptions discussed below). Therefore, decomposing scalp EEG data into component processes with *maximally* independent time courses should recover component processes whose scalp projection patterns should strongly resemble a single equivalent dipole [12], [13]. Here we show, for the first time, that linear blind source decomposition methods returning components with more independent time courses (ICA decompositions in particular) do in fact also return components with more nearly dipolar scalp maps, *even though none of the ICA/BSS algorithms we tested here either incorporate or take advantage of any biophysical or topographical information about electrode locations or source conduction patterns*.

### ICA and BSS algorithms

In the idealized case in which the source signals whose far field projections are mixed at the electrodes are truly independent – e.g., in decompositions of synthetic model data of unlimited length – many ICA algorithms may be expected to give equivalent results [27]. The most commonly reported algorithms to date (e.g., Infomax ICA, SOBI, and FastICA) are all able to extract several classes of non-brain artifacts [21]. Here, these algorithms extracted similar sources in several recognized brain and non-brain source categories (**Figures 2** **,** **3**). Nevertheless, our results show that the method used to separate ICA components does indeed affect the degree of temporal independence of the obtained components, as well as the number of such (near-dipolar) components that can be plausibly interpreted as representing the projection of a single cortical patch of near-synchronous field activity.

**Figure 4** shows that 18 of the 22 decomposition approaches we tested differed principally in only one dimension (not two) that tightly linked their degree of mutual information reduction and the number (or percentage) of their returned components with near-dipolar scalp maps that could represent the projection of locally synchronous field activity across a single cortical patch [11]. Our finding of a direct relationship between mutual information reduction and decomposition dipolarity is quite compatible with such a model of maximally independent EEG sources. The strongly *linearity* of this relationship across the 18 decompositions (from PCA to Infomax and AMICA) in **Figures 4** and **5** seems more difficult to predict. This result seems the more remarkable since the spatial relationships of the electrodes to each other or to the head were not entered into these algorithms but were, in effect, learned by them from the higher-order statistics of the temporal EEG dynamics.

We suggest that the linear relationship of mutual information reduction and dipolarity may only be explained by a direct physiological connection between the nature of source processes themselves and single equivalent dipole scalp projections. For the many component processes that originate in cortex, this may indeed reflect synchronous field activity across a cortical patch, while many non-brain (‘artifact’) EEG component projections may also resemble the projection of a single equivalent dipole within or at the surface of the head (e.g., scalp muscle activities, ocular artifacts, single-channel noise, electrocardiographic artifact). Distinguishing between near-dipolar brain and non-brain sources is typically straightforward based on their time courses, spectra, and the position of their equivalent dipole.

### Infomax ICA and AMICA

Interestingly, the five most efficient algorithms in our comparison all use natural gradient descent on approximations of the instantaneous data likelihood. Thus, it appears that this approach may indeed be optimal for efficient separation of component source processes from high-density EEG data [37]. The superior performance of the AMICA algorithm on these data may be explained by the fact that, in contrast to other infomax-related algorithms, AMICA attempts to model and use, for further refinement, each component's time course probability density function (PDF) as well as its spatial projection. By modeling the PDF of each component flexibly as a sum of extended Gaussians, AMICA may obtain better component separation than algorithms that assume one (or one of two) fixed parametric templates for each component PDF, as do standard or Extended Infomax [33]. When enough data are available, AMICA decomposition also scales well to high dimensions (e.g., to as many as 360 channels in our experience).

### Delay-dependent decompositions

Delay-dependent BSS algorithms SOBI, SOBIRO, SONS, AMUSE, icaMS, FOBI, EVD, and EVD-24 performed less well here, both in terms of mutual information reduction and dipolarity. Most of these algorithms rely on joint minimization of second-order correlations at multiple lags. Users of these algorithms may point out that their goal is not temporal independence *per se*, and thus the components these algorithms return may have other features of interest relevant to their individual objectives. Nevertheless, the components returned by the most tested of these algorithms, SOBI, tended to resemble components returned by instantaneous ICA (**Figures 1** and **2**).

### Second-order decompositions

The failure of PCA to return more than a very few near-dipolar components was not unexpected. The objective of Principal Component Analysis (PCA) is to lump together as much variance as possible into each successive principal component, whose scalp maps must then be orthogonal to all the others and therefore are not free to model a scalp source projection resembling a single dipole. The necessary orthogonality of principal component scalp maps guarantees that higher-order component scalp maps resemble checkerboards of various densities, while lowest-order principal components may be dominated by single large artifacts (e.g., eye blink artifacts) [38]. PCA components may thus be said to ‘lump’ together activity from many physiologically distinct, near-independent EEG sources so as to each contribute as much distinct *variance* to the data as possible. ICA algorithms, by contrast, attempt to‘split’ the raw data into maximally independent processes that each contribute as much distinct *information* to the mixed scalp channel signals as possible.

Sphering components, in particular, most often have stereotyped scalp maps consisting of a focal projection peaking at each respective data channel and thus resembling the projection of a radial equivalent dipole located beneath the central scalp channel (see Methods). This could possibly represent physiologically plausible projections of cortical EEG sources only if the cortex were smooth and unfolded, whereas the largest portion of the human cortex lies in its many sulci. Therefore, ICA decomposition approaches (including AMICA and infomax) that typically begin by sphering the data must progressively *reduce* the number of quasi-dipolar component maps they return (as may be seen in **Figure 3**), as the components adapt to the actual spatial projections of the actual still-more independent brain and non-brain sources in the data and as mutual independence among their time courses increases. Principal components themselves, as discussed above, are constrained to have orthogonal scalp maps and hence in general cannot be expected to resemble the output of a single cortical area. Yet in our assay PCA participated in the same linear trade-off between independence and near-dipolarity as 17 other BSS and ICA algorithms (**Figure 4A**), likely in part because the few largest components returned by all algorithms accounted for eye activity artifacts, whose projections to the scalp are approximately dipolar.

### Caveats and further comparisons

Our measure of overall decomposition ‘dipolarity,’ while clearly useful and informative (**Figure 4**), is also rather crude for at least two reasons. First, here we used only a spherical head model and common electrode coordinates to estimate best-fitting equivalent dipole models for the component maps and to estimate their residual variance from the actual component scalp maps. Better dipole fitting results should be obtained using boundary element method (BEM) or other anatomically more exact head models and more precisely co-registered electrode locations. More advanced (and complex) inverse approaches might estimate the location of the cortical source patches directly by building participant-specific electrical forward head models from participant magnetic resonance (MR) head images, which were not available for our participants [39]. Second, a more sensitive measure of physiological plausibility might explicitly model scalp muscles, ocular artifacts, and electrocardiographic artifact more precisely, and should allow for the possibility that a few independent sources are generated by synchronized activity from two strongly coupled cortical patches (or the two eyeballs). We do not see how our result should be expected to be compromised by such methodological improvements, though it does seem possible that the dipolarity threshold yielding the strongest linear relationship to MIR (6% residual variance, see **Figure 4B** and **C** insets) might be lower if more accurately individualized forward head models were used to compute it [39].

There might be multiple reasons for the inefficient results of the three ICA/BSS algorithms we did not include in **Figures 4B** and **4C** since they returned decompositions with less total mutual information reduction than second-order sphering. These include possible mismatches between our data and the default algorithm parameters we used. In particular, these parameters may have been optimized by their authors for decomposition of a few channels of idealized data rather than for realistic high-dimensional data. For this reason and for possible other future interest, we are making available (at http://sccn.ucsd.edu/eeglab/BSSComparison/) both the anonymized EEG datasets and the custom MATLAB (The Mathworks, Inc.) scripts we used to obtain the results in **Figure 4B**, with a hope that others may wish to run comparisons of optimized versions of these or other ICA/BSS algorithms to compare against the results presented here. Others may wish to propose figures of merit other than independence and dipolarity that bring out different types of utility for, e.g., decomposition methods that take into account dependence over time.

### Dependence remaining between maximally independent components

ICA decomposition has proven to be a highly useful approach for EEG data analysis, and our results here, as well as relevant reports on invasively acquired data [40], suggest that it may have substantial biological support. However, modeling cortical source activities as arising from exact synchrony across a cortical patch or phase-cone (as underlies decompositions such as AMICA) may have only first-order physiological model validity. Frequency-domain complex ICA decomposition methods may be able to more accurately model stereotyped radially expanding or contracting islands of synchronous activity (e.g., ‘phase cones’ or ‘avalanches’) associated with rhythmic EEG source activities, even when these have relatively small time-varying effects on the component process scalp projections [31], [41].

Our results show that other portions of the remaining mutual information between ICA components might reflect the still imperfect decomposition performance of even the best current ICA methods. Step-like changes in spatial source structure and/or transient or sustained periods of spatiotemporal dependence within one or more component subspaces are other possible sources of residual dependence. Spatiotemporal non-stationarities may include source processes that appear to travel long distances across cortex, such as sleep slow waves [42], K-complexes, and spindles [43] and some forms of epileptic seizures [40]. Still other changes in spatial source structure may accompany changes in subject cognitive state, task, or engagement. Thus, ICA methods that allow for modeling of spatial non-stationarity of the source configuration [31], [44], and/or more general convolutive process demixing [45], are of interest and might be tested in a manner resembling the present investigation. Although the PMI measure used here does not allow inference of causal relationships between component processes that exhibit residual dependence, methods based on Granger causality and transfer entropy might be used to examine this [46].

### Comparison to source isolation by response averaging

The ICA approach to EEG source identification contrasts with the long (and still) predominant approach of attempting to identify (only) those EEG sources active during peaks in averaged evoked potential epochs following abrupt onsets of experimentally presented sensory signals, on the assumption that these scalp maps sum the projection(s) of one or at most very few cortical source areas. Unfortunately, effects of sensory signals on the statistics of cortical field activities spread quite rapidly, concurrent with neural cross-talk and feedback between early sensory areas beginning about 30 ms [4], [47]. This makes it less likely that later peaks in average evoked potential waveforms represent the projection of activity from a single cortical source area, making spatial source filtering by response averaging and then ‘peak picking’ a relatively inefficient approach to isolating and locating individual EEG source signals.

Reduction of the EEG data by response averaging has the additional liability of discarding the large majority of the data, thus not allowing identification of the sources of the great majority of ongoing EEG activity that is not captured in ERP averages. The ICA approach by contrast, when favorably applied to suitable and sufficient data, allows identification of up to dozens of individual cortical source projections, both their maps and activity time courses, and their individual contributions to the ongoing (or trial averaged) data, making available a wider range of source and network level analyses while avoiding severe confounds produced by the unavoidable summation of their signals being broadly volume conducted from each brain and non-brain source to most of the scalp electrodes.

### Conclusion

Our results confirm that ICA and other BSS algorithms are capable of separating high-density EEG data into as many as dozens of processes with maximally independent time courses and near-dipolar scalp projections. Each unmixed maximally independent component process can be said to be a concurrently active source of *information* contributing to the data. Component processes with near-dipolar scalp projections may also represent physiologically distinct brain sources when and if they can be associated with field activity partially or fully synchronized across a cortical patch (or possibly across and between two anatomically well-connected patches). The tight connection between temporal independence and dipolarity demonstrated by our results is compatible with the hypothesis that cortical contributions to scalp EEG in large part sum far-field potentials from emergent islands or patches of near-synchronous cortical field activity. The activities of other (and sometimes many) maximally independent component processes are clearly generated, at least in large part, by non-brain sources typically identifiable as arising predominantly from eye blinks or saccades, scalp or neck muscle electromyographic activity, electrocardiographic contamination, line noise, single electrode noise, etc.

ICA decomposition transforms the problem of EEG analysis from analysis of the locally highly-correlated source signal mixtures recorded at the two-dimensional scalp surface to analysis of the time courses and spatial 3-D source distributions of maximally temporally independent data sources whose separate patterns of projection via volume conduction to the scalp sensors are given by the decomposition. More importantly for neuroscience, the enhanced signal-to-noise ratio of the unmixed independent source time courses of both brain and non-brain component processes allow study of relationships between multiple EEG source processes, behavior, and subject experience through a collection of single trials and/or in the continuous data record. Further, the separated component scalp maps greatly simplify the process of identifying the cortical patch (or non-brain source) involved in generating each identified source activity using a suitable biophysical inverse method.

Here we have shown, first, that although some ICA and BSS algorithms return larger numbers of EEG components with more nearly dipolar scalp maps than others, they appear to identify similar, biologically plausible brain and non-brain component processes. Further, we have shown that for many ICA and BSS algorithms applied to our data, their degree of efficiency in reducing mutual information among the resulting component time courses is positively correlated with the number of biologically plausible near-dipolar components they return. Moreover, for 18 such algorithms (including algorithms as diverse as PCA, SOBI, FastICA, JADE, infomax ICA, and AMICA), this relationship appears to be, on average, linear, a result that invites deeper study since the methods used by these algorithms appear on the surface diverse and dipolarity and MIR would seem to have no inherently simple numerical relationship.

To invite comparisons of the methods tested with other linear decomposition techniques, we are making the anonymized EEG data and custom MATLAB analysis and plotting scripts available for this purpose (see Methods). We hope these results may serve to increase the acceptance of the utility of ICA methods for (a) separating the statistical question of *what* EEG source activity time courses compose the data record from the biophysical inverse problem of finding *where* these source activities take place, and (b) separating the spatial source projection pattern for each identified source signal, thus simplifying the biophysical inverse problem for sources in the whole data, rather than only in limited response averages drawn from it. We hope that extracting source-level information from high-density EEG data by ICA decomposition brings closer the goal of developing high-density EEG imaging into a true functional 3-D cortical imaging modality, with high temporal resolution and spatial resolution adequate for studying distributed macroscopic cortical brain processes supporting both normal and abnormal behavior and experience.

## Methods

### Ethics Statement

Human subject data presented in this article have been acquired under an experimental protocol approved by an Institutional Review Board of University of California San Diego. Written consent was obtained from each subject.

### Participant task

Fourteen volunteer participants performed a visual working memory task [25]. At the beginning of each trial a central fixation symbol was presented for 5 sec. A series of eight single letters, 3–7 of which were black (to be memorized) and the rest green (to be ignored), were then presented for 1.2 s with 200-ms gaps. Following these, a dash appeared on the screen for 2–4 s to signal a memory maintenance period during which the participant was to retain the sequence of memorized letters until a (red) probe letter was presented. The participant then had to press one of two buttons with their dominant hand (index finger or thumb) to indicate whether or not the probe letter was part of the memorized letter set. Auditory feedback 400 ms after the button press informed the participant whether their answer was correct or not. The next trial began when the participant pressed another button. Each participant performed 100–150 task trials - see Onton et al. [25] for additional details and event-related analyses. We did not consider event-related analysis of the data in the present study.

### EEG data

EEG data were collected from 71 channels (69 scalp and 2 periocular electrodes, all referred to right mastoid) at a sampling rate of 250 Hz with an analog pass band of 0.01 to 100 Hz (SA Instrumentation, San Diego). Input impedances were brought under 5 kΩ by careful scalp preparation. We initially selected data from 14 out of 23 participants based on the perceived quality of the original ICA decompositions under visual inspection (7 males, 7 females, mean age 25±6.5 years). Of these, we informally judged seven to give ‘better’ extended infomax ICA decompositions (defined by a relatively large number of component scalp maps that resembled the projection of a single dipole), and seven to give ‘poorer’ ICA decompositions (with fewer such component maps). For one of the participants with an unusually ‘poor’ ICA decomposition, for unknown reasons all ICA/BSS algorithms failed to substantially reduce mutual information from the level of the raw scalp channels. Results for this data set were also unreliable across algorithms, so data from this participant were excluded, leaving data sets from 13 participants to be used in the comparisons.

### Data analysis

Data were analyzed by custom MATLAB scripts built on the open source EEGLAB toolbox [48]. Continuous data were first high-pass filtered above 0.5 Hz using a FIR filter. Data epochs were then selected from 700 ms before to 700 ms after each letter presentation. The mean channel values were removed from each epoch, and between 1 and 16 noisy data epochs were removed by visual inspection before ICA decomposition. Criteria for epoch removal were the presence of high-amplitude, high-frequency abnormalities (such as those accompanying occasional coughs, sneezes, jaw clenching, etc.). The total number of data samples in each dataset was between 296,000 and 315,000.

### Algorithm selection

We tested a total of 22 linear decomposition algorithms, 20 ICA or BSS algorithms plus principal component analysis (PCA) and PCA-related data whitening or sphering. MATLAB code for the ICA algorithms we used can be downloaded from the Internet (see Table 1). The selected algorithms all perform complete decompositions in which the number of returned components is equal to the number of channels:
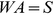
(1)where 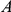 is the data matrix of size (*number of channels* by *number of time points*), 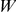 is an unmixing matrix of size (*number of ICA components* by *number of channels*), and 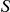 is the ICA component activation time courses of size (*number of ICA components* by *number of time points*).

### Natural gradient approach

ICA and BSS algorithms learn the unmixing weight matrix that makes the resulting component time courses or activations as temporally independent from each other as possible. However, the approach of each algorithm to estimating and/or approaching this independence is different. AMICA [33], Extended Infomax [27], Infomax [28], Pearson ICA [49], and ERICA [50] belong to the class of natural gradient ICA algorithms [37], differing only in the way they estimate the component probability distributions. These algorithms are highlighted in yellow in **Figure 4**.

### Second-order time-delay approach

SOBI [30] is a second-order BSS method that attempts to simultaneously reduce a large number of time-delay correlations (by default, 100) between the source activities. SOBIRO (a variant of SOBI using a robust orthogonalization method), SONS, AMUSE, icaMS, FOBI, EVD, and EVD 24 all use time-delay covariance matrices [51]. For all these algorithms, we selected the default time delays as implemented in the downloaded software implementations. These algorithms are highlighted in blue in **Figure 4**.

### Other methods

Other algorithms including (so-called) FastICA maximize the negentropy of their component distributions or their fourth-order cumulants (e.g., JADE; JADE optimized) [29], [52]. Extensive documentation of these algorithms is available (e.g., [28], [29], [51]). We used the software default parameters; it is possible that better ICA decompositions might have been obtained in some cases using other parameter choices. These algorithms are highlighted in pink in **Figure 4**.

### Principal Component Analysis (PCA)

ICA and BSS algorithms differ from principal component analysis (PCA) in that they identify sources of distinct *information* in the data instead of, like PCA, characterizing orthogonal directions of maximal *variance* in the data. Thereby ICA can bypass PCA's spatial orthogonality constraint that invariably gives a complex checkerboard appearance to scalp topographies of high-order PCA components [38]. We included PCA in our assay because it has been used to decompose EEG and ERP data [53], and because of its relation to sphering, which is often used to preprocess data before applying ICA decomposition. PCA is highlighted in green in **Figure 4**.

### Sphering

Sphering decorrelates the component pair time courses while leaving each component scalp map centered on an original scalp channel. It is equivalent to rotating the data into their principal component basis, equating data variance along all principal axes (thus ‘whitening’ the data), and then rotating the data back to their original channel basis [28]. Any rotation of the whitened data by a rotation matrix 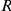 will retain unit variance in all directions. Choosing 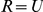, we get 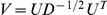. Since 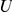 is orthonormal, we have 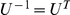. Thus transforming by 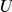 reverses or undoes the initial rotation 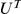 in which the data were projected onto the eigenvectors. The whitened data (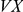) transformed by 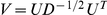 then are not the same as the original data; in particular, the data now have unit variance in all directions. Since sphering is a linear spatial transform of the data, the columns of the inverse sphering ‘mixing’ matrix (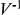) contain the sphering component topographies (see examples in **Figure 1**, bottom row). Note that these are centered on successive single channels in the submitted channel list.

### Measuring entropy and mutual information

Independence of a set of random variables can be measured by the mutual information. Mutual information is defined in terms of the entropy (a measure of the degree of randomness or unpredictability) of the data, which for a continuous random vector 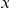, is called the *differential entropy*, and is defined by
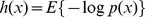
(2)where 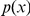 is the probability density function of the random vector 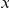.

The *mutual information* between two random variables 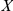 and 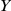 can be defined as the difference between the sum of the individual (marginal) entropies of 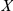 and 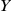, and the joint entropy of 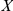 and 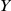, 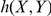


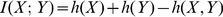
(3)Similarly the mutual information in, or among, the components of a random vector 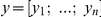 is defined by
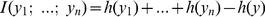
(4)


The mutual information is always non-negative since the joint entropy of a random vector is always less than or equal to the sum of the marginal entropies, with equality only if the components of the random vector are independent. Larger mutual information indicates that the entropy or “uncertainty” in the joint distribution of 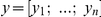 is significantly lower than the entropy in the factorial distribution (the product of the marginals that would result if the random variables were independent). That is, there is some dependence in the component time courses that makes the value of the vector 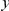 less uncertain when considered as a multi-dimensional whole than when each of its components are considered separately (e.g., ignoring any dependence-derived information in the multi-dimensional distribution about which *combinations* of component values are more or less likely than the individual component values would in themselves dictate).

### Mutual information and linear transforms

For ICA analysis, the observed EEG data are modeled as an independent and identically distributed (i.i.d.) realization of a vector time series 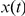, the observed linear mixture of a set of 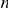 sources 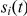 activating the corresponding component projection topographies 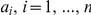, so that
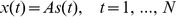
(5)ICA decomposition attempts to estimate the sources by learning the un-mixing matrix 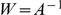 such that
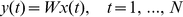
(6)where 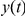 is equivalent to 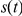 except for permutation and scaling of the components – operations that do not change the degree of dependence or mutual information among the components. The strategy used by many ICA algorithms involves estimating 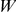 so as to minimize the mutual information 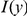 of the resulting component signals. The actual cost function employed depends on how the component joint density function is approximated.

In cumulant ICA methods, the source densities are expanded in terms of cumulants (e.g., mean, variance, kurtosis) that are estimated empirically from the partially unmixed data as the un-mixing matrix is optimized [54], [55]. The source densities are generally taken to be members of a parametric family of distributions (or a quasi-parametric family in the case of mixture models), and the density parameters are optimized along with the un-mixing matrix to maximize the likelihood [33]. In the quasi-parametric case, with the optimization being viewed as over all possible source densities, this amounts to minimization of the mutual information itself.

### Component Mutual Information

For the linear transformation 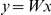, the entropy of the continuous vector variable y is given by
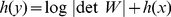
(7)By (3), the mutual information of the transformed data, 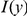, is then
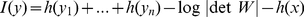
(8)Since 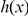, the joint entropy of the data, 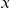, is independent of 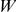, the minimization of the mutual information over 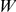 (and possibly over parameters of the source density models 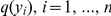 essentially consists of minimizing the sum of the marginal entropies, 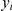, minus a term involving the determinant of 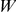. Thus ICA algorithms following this approach must model only one-dimensional densities, either parametrically from the data [33] or using cumulant expressions [28], [29]. Most of the algorithms we use here also apply sphering (or some more general whitening procedure) as a pre-processing step to remove second-order dependency (correlations) between the (sphered) channel signals. Details on the entropy calculations are provided in the following section.

### Pairwise Mutual Information (PMI)

The pairwise mutual information matrix 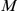 for the set of time series 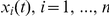, considered as 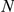 samples of the random variables 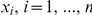, is defined by
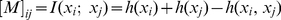
(9)


We estimate the entropy using the usual binning method, where histograms and a simple Riemann approximation to the integrals are used to compute the entropies. This approach is generally suitable for large sample sizes like those encountered in EEG. Approximate asymptotic variance of the estimate is also available in terms of number of bins and number of samples, allowing us to assess the statistical significance of the results.

We choose a fixed number of bins, 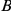, for all univariate random variables, and construct the 2-D histograms using 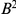 bins, using the same marginal bin endpoints as used for the one-dimensional histograms. Specifically, let the one-dimensional histogram of data 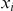 be denoted 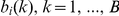, where 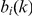 is the number of time points 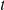 for which the value of 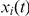 is in the *k*th bin. The estimate of the continuous one-dimensional density 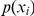 is taken to be 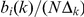 over the *k*th bin, where 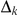 is the size of the *k*th bin, and 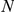 is the total number of time points. This makes the continuous density integrate to one in the Riemann approximation,
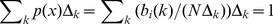
(10)Since we use 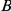 bins distributed over the maximum and minimum values of the time series, the bin size 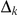 is 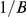 for all 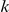. The estimate of the one-dimensional marginal entropy is then given by,
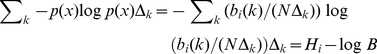
(11)where 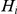 is the discrete entropy of the 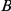-dimensional discrete probability distribution defined by 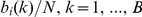.

The two dimensional joint entropy is estimated similarly, with the joint density of 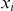 and 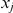 taken to have the constant value 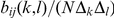 over the 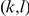 bin, where 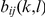 is the number of time points for which 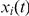 is in the *k*th bin and 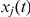 is in the *l*th bin. The estimate of the joint entropy is then,
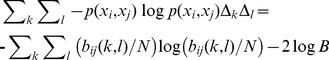
(12)We define the discrete joint “bin entropy” as,
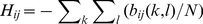
(13)Now, for the estimate of the mutual information between *x_i_* and *x_j_*, we have,
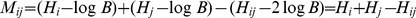
(14)Thus the estimate of the mutual information does not depend on the bin size, only on the bin entropies. This result does not depend on the fact that equal bin sizes were used. Even with arbitrary bin sizes, the bin size terms cancel. Note that total PMI (the sum of PMI for all 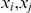 pairs) may be larger than MI when higher-order dependencies are present in the data.

The approximate entropy estimator bias is given by
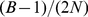
(15)where 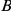 is the number of bins and 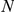 is the number of samples [56]. If we define 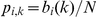, and 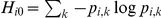, then the approximate variance of the bias corrected entropy estimate for source 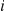 is
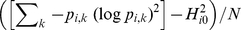
(16)Since the PMI and MIR estimates are expressed in terms of sums of entropies, their variances can be calculated by summing the appropriate entropy variances. Note that mutual information estimates were bias corrected. Error bars are not shown in any of the figures because they were not visible at the scale of the plots. A caveat to the validity of the estimates is that they assume stationarity of data, which does not necessarily hold in the case of EEG data. However the measures can still be used to assess relative performance of unmixing matrices, as well as to assess the co-variation of dipolarity and other measures with mutual information reduction (MIR).

### Mutual Information Reduction (MIR)

The reduction in mutual information achieved by a potential ICA matrix W can be calculated using only one-dimensional density models. Specifically, the *mutual information reduction* (MIR), i.e. the amount of mutual information removed from set of channels, is given by,
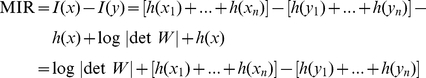
(17)The MIR thus depends only on the difference between the sums of the marginal entropies of 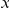 and 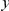 and on the (log) determinant of 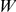. The marginal entropies involved are readily estimated using histograms of the channel and estimated source data (as in the preceding section on direct estimation of pairwise mutual information). ICA attempts to minimize mutual information in the estimated sources, 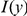, and thus to maximize MIR. Thus, MIR for an ICA algorithm is expected to be positive for a given dataset, and algorithms reducing the mutual information more, thereby producing more independent sources, will have higher MIR.

### Equivalent dipole modeling

After computing all 22 decompositions for each of the 13 EEG datasets, we localized a best-fitting single equivalent dipole corresponding to each returned component using a single equivalent dipole in a best-fitting spherical four-shell model head (radius: 71, 72, 79, 85 mm; shell conductances: 0.33, 0.0042, 1, 0.33 µS) using the DIPFIT plug-in (version 1.02) in the EEGLAB toolbox (version 4.515) [48]. To avoid errors based on the simplistic head model we used in the computations, scalp map values for two electrodes sited near the eyes were excluded from dipole fitting. Note that modeling each component map with a single dipole is somewhat idealistic, since in particular some ICA components represent apparently bilateral synchronous source activities (e.g., the component maps in the third column of **Figure 1**). However, brain components clearly warranting a dual-dipole model appeared to be rare (approximately one per decomposition), as we typically find in other decompositions of more than 32 data channels. Components accounting for most electro-oculographic (EOG) artifacts should also be modeled using two (peri-ocular) dipoles, but as the eyes are relatively close together and accurate forward modeling the front of the skull is difficult, the additional errors introduced by using single dipole models for EOG components is not large.

### Data and methods available

The 13 anonymized data sets used in the analysis are available for download (http://sccn.ucsd.edu/wiki/BSSComparison) together with a ‘README’ file and MATLAB scripts using EEGLAB functions (http://sccn.ucsd.edu/eeglab) that perform the algorithm comparisons including computation of mutual information reduction (MIR) and dipolarity (dipole fitting). Plotting scripts are also included for reconstructing **Figure 4B** and adding a new data point for a new decomposition algorithm. Using these scripts and the referenced EEGLAB functions (http://sccn.ucsd.edu/eeglab), interested readers may compare MIR and dipolarity of any other linear decompositions of these data, and may compare results of an algorithm of their choice with our results as applied to the same data sets. The authors invite readers to send us results of these comparisons for inclusion on the web page above, following instructions contained in the README file.
